# Learning curve of radiology residents during training in fluoroscopy-guided facet joint injections

**DOI:** 10.1590/0100-3984.2015.0176

**Published:** 2017

**Authors:** Tiago Rocha Dias, João de Deus da Costa Alves Junior, Nitamar Abdala

**Affiliations:** 1 MD, Radiologist, Research Student in Radiology, Department of Diagnostic Imaging, Escola Paulista de Medicina da Universidade Federal de São Paulo (EPM-Unifesp), São Paulo, SP, Brazil.; 2 MD, Interventional Neuroradiologist, Research Student in Radiology, Department of Diagnostic Imaging, Escola Paulista de Medicina da Universidade Federal de São Paulo (EPM-Unifesp), São Paulo, SP, Brazil.; 3 Full Professor of Radiology, Head of the Department of Diagnostic Imaging, Escola Paulista de Medicina da Universidade Federal de São Paulo (EPM-Unifesp), São Paulo, SP, Brazil.

**Keywords:** Zygapophyseal joint, Injections, intra-articular, Injections, spinal, Learning curve, Models, anatomic, Radiology, interventional

## Abstract

**Objective:**

To develop a simulator for training in fluoroscopy-guided facet joint injections and to evaluate the learning curve for this procedure among radiology residents.

**Materials and Methods:**

Using a human lumbar spine as a model, we manufactured five lumbar vertebrae made of methacrylate and plaster. These vertebrae were assembled in order to create an anatomical model of the lumbar spine. We used a silicon casing to simulate the paravertebral muscles. The model was placed into the trunk of a plastic mannequin. From a group of radiology residents, we recruited 12 volunteers. During simulation-based training sessions, each student carried out 16 lumbar facet injections. We used three parameters to assess the learning curves: procedure time; fluoroscopy time; and quality of the procedure, as defined by the positioning of the needle.

**Results:**

During the training, the learning curves of all the students showed improvement in terms of the procedure and fluoroscopy times. The quality of the procedure parameter also showed improvement, as evidenced by a decrease in the number of inappropriate injections.

**Conclusion:**

We present a simple, inexpensive simulation model for training in facet joint injections. The learning curves of our trainees using the simulator showed improvement in all of the parameters assessed.

## Abstract (pt)

**Objetivo:**

Desenvolver um simulador para treinamento em punção de articulações facetárias guiada por fluoroscopia e avaliar a curva de aprendizado neste procedimento em um grupo de residentes de radiologia.

**Materiais e Métodos:**

Tomando uma coluna lombar humana como modelo, desenvolvemos cinco vértebras lombares feitas de metacrilato e gesso. Essas vértebras foram combinadas para formar um modelo anatômico de coluna lombar. Utilizamos um invólucro de silicone para simular a musculatura paravertebral. O modelo foi colocado dentro do tronco de um manequim de plástico. Recrutamos 12 voluntários dentre residentes de radiologia de nosso departamento. Cada aluno realizou 16 punções de articulações facetárias em nosso simulador em uma única sessão de treinamento. Usamos três parâmetros para avaliar as curvas de aprendizado: tempo de procedimento, tempo de fluoroscopia e qualidade do procedimento, definida pelo posicionamento da agulha.

**Resultados:**

As curvas de aprendizado de todos os estudantes mostraram melhora nos tempos de procedimento e fluoroscopia com o treinamento. O parâmetro de qualidade do procedimento também mostrou melhora, definida por decréscimo no número de punções inadequadas.

**Conclusão:**

Apresentamos um modelo simulador simples e de baixo custo para treinamento em punção de articulações facetárias. As curvas de aprendizado de nossos estudantes mostraram melhora em todos os parâmetros avaliados.

## INTRODUCTION

Facet joint injections are often used in order to treat or diagnose diseases of those same joints^(1-4)^. They can be guided by fluoroscopy, computed tomography, ultrasound, or magnetic resonance imaging^(1,2,5)^. Fluoroscopy is widely available and is broadly used in order to guide facet joint injections. Fluoroscopy has the advantage of reducing the procedure time^(1,5,6)^.

Learning medical techniques usually requires many hours of training. Deliberate practice and repetition are essential for obtaining and maintaining proficiency^(7,8)^. Learning curves are graphical representations of improvement in the performance of a certain task over time (i.e., with experience). The performance of medical techniques can be assessed by using variables such as procedure time and number of complications, whereas experience can be assessed by using other variables, such as number of repetitions and hours of training^(9,10)^. Learning curves are often used in order to evaluate medical training. They provide information regarding the variables that affect the learning process and may assist in curriculum planning^(10,11)^. They have been extensively used in the evaluation of surgical techniques and minimally invasive procedures^(11-13)^.

In medical education, the traditional model of learning, based on observing and participating in real procedures, has limitations, such as the limited number of procedures available for repetition and potential harm to patients^(14)^. Simulators have the potential to improve ability and confidence in complex and risky procedures, allowing students to make mistakes during the training without harm to patients. They also allow objective assessment of learning parameters^(14,15)^. Abilities acquired in simulators may lead to improved performance in real procedures^(11,16)^. The use of simulators gained great importance in the surgical field after the advent of laparoscopic surgery^(7,14,17)^. Subsequently, their use has spread to other fields, including interventional radiology and neuroradiology^(15,17-22)^.

The objective of this study was to develop a simple, inexpensive simulator for training in fluoroscopy-guided facet joint injections that could be used in interventional radiology training programs, as well as to evaluate the learning curve for this procedure among radiology residents.

## MATERIALS AND METHODS

### Development of the simulation model

We refined a simulation model previously created^(18)^. Five vertebrae were developed based on the lumbar spine of a human cadaver. To make the vertebrae, a silicon mold based on each vertebra was filled with sponges soaked in methacrylate and plaster, simulating the bone marrow. An external layer of methacrylate and plaster was added to simulate the cortex.

**Figure 1:**
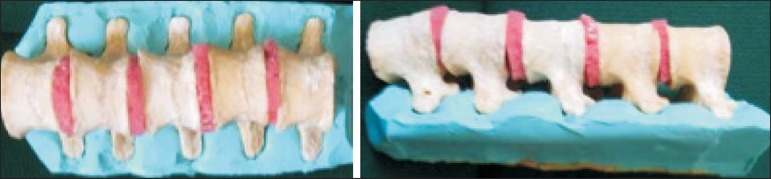
Spine model composed of five lumbar vertebrae placed within a silicon mold.

The five vertebrae were connected by 1.5 cm thick fragments of sponge soaked in polymethylsiloxane and silica, simulating intervertebral discs. The facet joint spaces were also filled with polymethylsiloxane and silica. We developed a silicon mold with a tactile consistency similar to that of the paravertebral muscles. The spine model was placed inside that mold (Figure 1).

**Figure 2:**
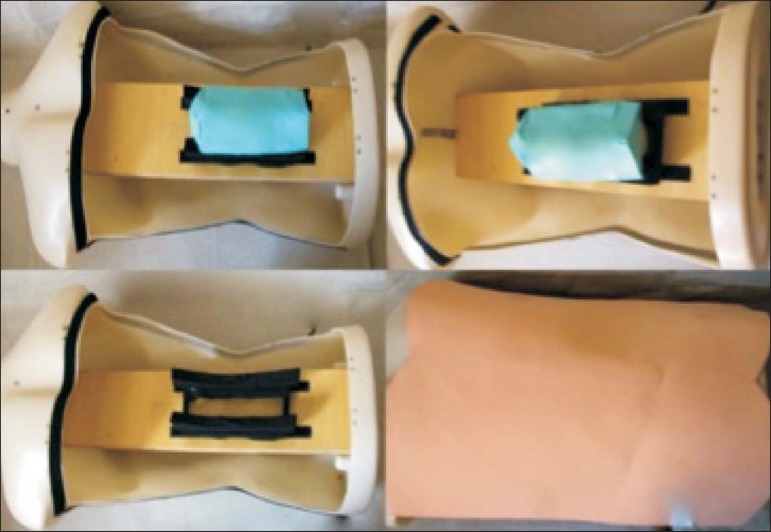
Assembling the model.

We used a plastic mannequin to simulate the human trunk. We removed its posterior surface. Inside it, we built a wooden plate along the longitudinal axis of the trunk, and fixated the plate with screws. On the surface of the plate, we placed two parallel strips of ethylene-vinyl acetate, 4.0 cm away from each other. Each strip was 30 cm long and 2.0 cm wide, with a height of 4.0 cm in the middle and 5.0 cm at the extremities. The spine model was placed on these strips. We covered the posterior surface of the mannequin with a 0.5 cm sheet of ethylene-vinyl acetate (Figure 2).

According to an experienced radiologist, the model simulated closely the anatomy and tactile consistency of real patients.

### Recruitment of volunteers

Twelve first-, second-, or third-year radiology residents volunteered for training using our simulator, and all were accepted for inclusion in the study. None had previous experience with facet joint injections. The training sessions were conducted under the supervision of a qualified, experienced radiologist. An experienced radiologist also volunteered to test the simulator. For every volunteer, the individual radiation dose received was within the acceptable range according to local legislation. The study was approved by the Ethics Review Committee of the Federal University of São Paulo - Paulista School of Medicine, on May 30, 2008.

### Instruments and procedure

The procedures were performed at the Hospital São Paulo/Paulista School of Medicine. We employed a digital subtraction angiography system (Integris V5000; Philips Medical Systems, Eindhoven, Netherlands), using lead aprons and thyroid collars for radiation protection.

Prior to attempting the injections, the volunteers received a theoretical explanation and watched a member of our team demonstrate the procedure using the simulator. Each volunteer carried out 16 injections in the simulator, in a single training session, divided in two phases. In the first phase, the volunteers carried out the injections at the L1-L2 to L4-L5 levels, first on the left side and then on the right side. In the second phase, they repeated the injections at the same levels, in the same order.

The procedures began with the model, X-ray tube, and table placed in standard neutral positions. The volunteers were allowed to move the tube, table, and model freely during the procedure but were required to return them to their original positions after completing each injection. Between injections, the supervisor provided feedback, pointing out mistakes and offering tips to improve performance.

We used the direct intra-articular injection approach. The simulator remained in ventral decubitus, and the X-ray tube was angulated obliquely to demonstrate the articular space of the facet joint. The goal of the procedure was to insert a 22-gauge needle directly into the articular space. Plain films were acquired in oblique, lateral, and frontal incidences to document the position of the needle.

### Parameters evaluated

**Figure 3:**
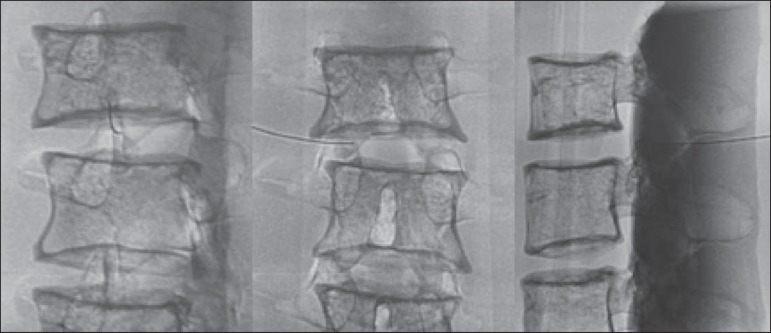
Radiographic appearance of an appropriate procedure.

We evaluated three parameters: procedure time; fluoroscopy time; and qualitative classification of the procedure, based on the positioning of the needle. Procedure time was measured in seconds using a chronometer attached to the fluoroscopy machine. It consisted of the entire time taken to carry out the injection. Fluoroscopy time was measured in minutes, as provided by the fluoroscopy machine. An experienced radiologist made a qualitative assessment of the procedures, based on the plain films. The radiologist was blinded to the order of the injections and to the names of the performing volunteers. The procedures were classified as either appropriate or inappropriate. Procedures in which the final position of the needle was either intra-articular or adjacent to the joint (periarticular) were considered appropriate (Figure 3). Those in which the final position of the needle was far away from the articular space were considered inappropriate.

### Statistical analysis

The learning process was described graphically using dispersion diagrams for the procedure time and fluoroscopy time parameters. Bar charts were used in order to illustrate the qualitative classification of the procedures.

To assess improvement in the procedure time and fluoroscopy time parameters, we adopted the Wright model for learning curves, also known as the potential model. In that model, the mathematical expression of the learning curve is:

$$y=\alpha \times x^{\beta }$$

where *y* is the procedure time (or fluoroscopy time) for the nth procedure; α is the estimated time to perform the first procedure; *x* is the cumulative number of procedures performed; β is the angle, or learning, parameter (representing the slope of the learning curve). The value of β is mathematically estimated and is related to the learning rate of the student. From that estimation, we calculated the learning rate as 2^β^ (expressed as a percentage).

We chose the Wright model because it is the most widely used for learning curves, and because it was the one that best fit our data, according to the coefficient of determination (R^2^).

We first adjusted the potential model separately for each volunteer. The estimations obtained were summarized in means, standard deviations, and coefficients of variation. To obtain a global evaluation and perform a statistical analysis of the effect of the training on the procedure time and fluoroscopy time parameters, we used a mixed linear model in which all data were analyzed together, considering the correlation (dependency) among the observations of each single individual. We assumed a structure of autoregressive correlation, which assumes a stronger correlation among subsequent procedures.

For the qualitative analysis of the procedures, we applied the binomial model for dependent data to assess the potential increase in the chance of carrying out an appropriate injection as the training progresses.

We sought to simplify the analysis by considering the training in two phases. Phase 1 comprised procedures 1 through 8. Phase 2 comprised procedures 9 through 16. We compared the two phases in terms of the number of appropriate injections, using the Wilcoxon signed-rank test.

## RESULTS

### Procedure time analysis

**Figure 4:**
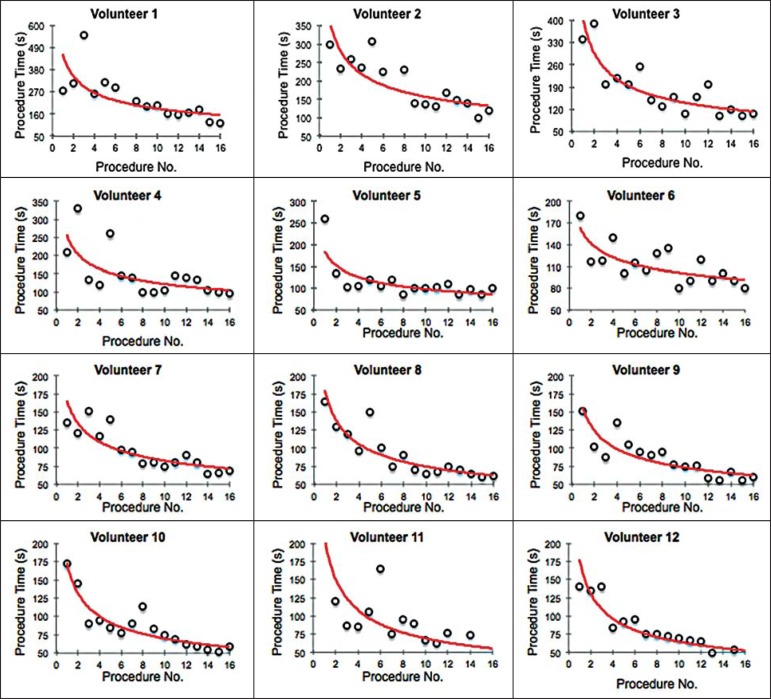
Graphical representation of the procedure time (in seconds) for the injections carried out.

Figure 4 shows the evolution of the procedure time over the course of the 16 injections performed by each of the 12 volunteers. There was progressive decrease in procedure time during the training. The estimations obtained after the adjustment of the potential model are presented in Table 1. For all of the volunteers, the inclination parameter was negative and statistically significant (different from 0). The mean learning rate was 78%, ranging from 71.7% to 87.1%. According to the general model, the estimated procedure time for the first injection was 208.5 s (95% confidence interval [95% CI]: 165.7 to 262.4) and the slope of the curve was 0.337 (standard error of 0.052), corresponding to a learning rate of 79.17%. The *p*-value for the inclination was less than 0.001. The experienced radiologist performed injections with procedure times ranging from 39 s to 50 s.

**Table 1:** Estimates obtained in the potential model for procedure time.

	Fluoroscopy time estimated for the first injection			Slope of the curve			Quality of the adjustment	
Volunteer	α	Standard error		β	Standard error		R^2^	Learning rate
1	2.999	0.802		-0.293	0.124		0.283	81.6%
2	1.862	0.441		-0.335*	0.114		0.397	79.3%
3	1.640	0.299		-0.308^†^	0.088		0.465	80.8%
4	1.315	0.415		-0.319	0.153		0.250	80.2%
5	1.086	0.187		-0.262^†^	0.083		0.413	83.4%
6	0.818	0.192		-0.253*	0.113		0.262	83.9%
7	1.215	0.185		-0.374‡	0.074		0.648	77.2%
8	1.088	0.153		-0.354‡	0.068		0.657	78.2%
9	0.898	0.102		-0.359‡	0.055		0.754	78.0%
10	1.165	0.113		-0.355‡	0.047		0.803	78.2%
11	1.494	0.254		-0.545‡	0.082		0.757	68.5%
12	0.942	0.087		-0.362‡	0.045		0.824	77.8%
Mean	1.38			-0.34			0.54	78.9%
Standard deviation	0.60			0.07			0.22	3.9%
Coefficient of variation	43.38%			21.81%			40.73%	5.0%

* p ≤ 0.001 and

† p ≤ 0.003 for the hypothesis β = 0.

### Fluoroscopy time analysis

**Figure 5:**
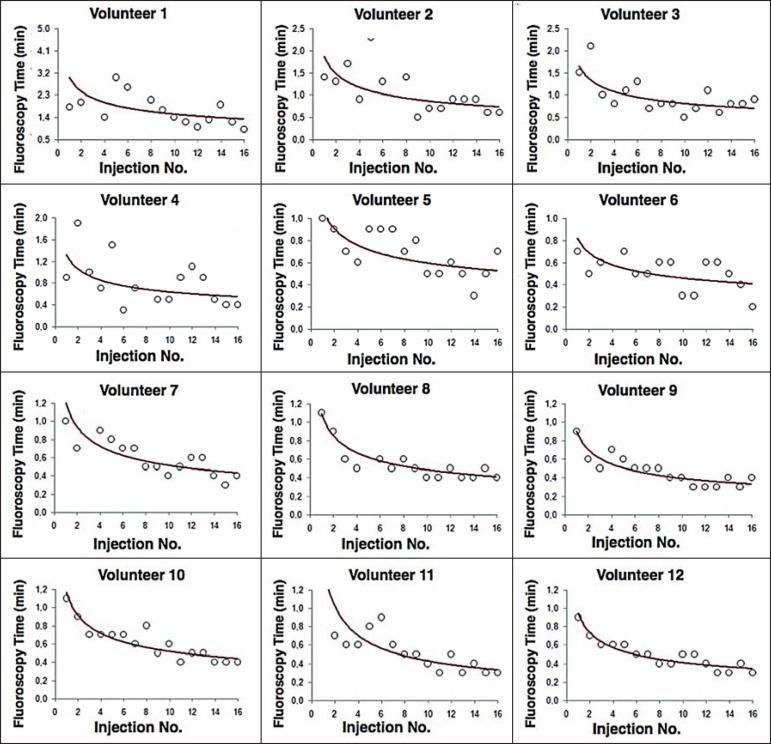
Graphical representation of the fluoroscopy time (in minutes) of the injections carried out. Equations represent the adjustment of the Wright model. *y*, fluoroscopy time; *x*, cumulative number of injections; *R^2^* = coefficient of determination (quality of the adjustment).

All volunteers showed progressive improvement in the fluoroscopy time parameter (Figure 5). For 11 of the 12 volunteers, the inclination parameter was statistically significant (different from zero). The mean learning rate was 79%, ranging from 68.5% to 83.9% (Table 2). The general model produced an estimate of 78 s for the fluoroscopy time of the first injection (95% CI: 0.39 to 1.53), an inclination of the learning curve of -0.308 (95% CI: -0.186 to -00.431) and a learning rate of 80.7%. The p-value for the inclination was less than 0,001, confirming the significant decrease in fluoroscopy time. The experienced radiologist performed all the procedures in the same fluoroscopy time, 0.3 min.

**Table 2:** Estimates obtained in the potential model for fluoroscopy time.

	Fluoroscopy time estimated for the first injection			Slope of the curve			Quality of the adjustment	
Volunteer	α	Standard error		β	Standard error		R^2^	Learning rate
1	2.999	0.802		-0.293	0.124		0.283	81.6%
2	1.862	0.441		-0.335*	0.114		0.397	79.3%
3	1.640	0.299		-0.308^†^	0.088		0.465	80.8%
4	1.315	0.415		-0.319	0.153		0.250	80.2%
5	1.086	0.187		-0.262^†^	0.083		0.413	83.4%
6	0.818	0.192		-0.253*	0.113		0.262	83.9%
7	1.215	0.185		-0.374^‡^	0.074		0.648	77.2%
8	1.088	0.153		-0.354^‡^	0.068		0.657	78.2%
9	0.898	0.102		-0.359^‡^	0.055		0.754	78.0%
10	1.165	0.113		-0.355^‡^	0.047		0.803	78.2%
11	1.494	0.254		-0.545^‡^	0.082		0.757	68.5%
12	0.942	0.087		-0.362^‡^	0.045		0.824	77.8%
Mean	1.38			-0.34			0.54	78.9%
Standard deviation	0.60			0.07			0.22	3.9%
Coefficient of variation	43.38%			21.81%			40.73%	5.0%

* p ≤ 0.05,

† p ≤ 0.01 and

‡ p ≤ 0.001 for the hypothesis β = 0.

### Qualitative analysis of the procedures

When we applied the binomial model for dependent data, we observed that the training had a statistically significant effect (*p* < 0.001), indicating that as the procedures are performed, the chance of carrying out an appropriate injection significantly increases (odds ratio = 1.62; 95% CI: 1.28 to 2.05). According to this model, at each new procedure, the chance of carrying out an appropriate injection increases 62%.

**Figure 6:**
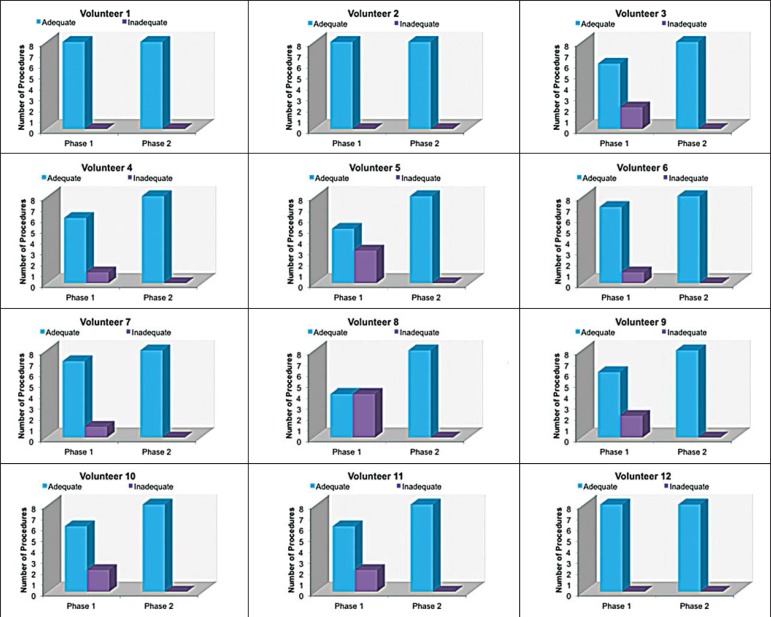
Graphical representation of the absolute frequencies of appropriate and inappropriate procedures during the two phases of the training.

We compared the two training phases in terms of the numbers of appropriate and inappropriate injections. Figure 6 shows the number of appropriate injections carried out by each volunteer in each phase. The Wilcoxon signed-rank test showed a statistically significant difference between the two phases (*p* = 0.007). Notably, all of the injections carried out in phase 2 were considered appropriate. All of the injections carried out by the experienced radiologist were also considered appropriate.

## DISCUSSION

The learning curves of the volunteers show that there was a progressive decrease in procedure time during the training. That decrease was statistically significant, indicating that learning occurred. The procedure time parameter is widely used in determining learning curves in medicine^(8-11,14,17,23)^. Other parameters used in learning curves for surgical and interventional procedures, such as the probability of complications and mortality, are difficult to measure, because of the low prevalence of the events of interest, and might not be directly related to the technique employed in the procedure. Procedure time has the advantage of providing objective and easily quantifiable information^(9)^. Although the performance of a procedure in a shorter time does not necessarily imply greater quality of the procedure^(9)^, it usually reflects the gains in experience and confidence acquired with practice^(24,25)^. A decrease in procedure time is often accompanied by a reduced number of complications^(26)^.

Fluoroscopy time has also been used previously in learning curves^(24,27)^. To minimize patient and operator radiation exposure^(28)^, fluoroscopy time should be as low as possible. A decrease in fluoroscopy time naturally follows a decrease in procedure time but might also be related to other factors, such as the attention given to avoiding the unnecessary use of fluoroscopy, together with the use of palpation and other techniques to reduce fluoroscopy time^(28)^. In the present study, the learning curves of the volunteers showed a progressive decrease in fluoroscopy time, particularly at the beginning of the training. Other studies assessing fluoroscopy time in various procedures have reported similar results^(24,27)^.

In a simulator, it is not possible to assess some parameters that are directly related to the technical quality of the procedures, such as procedure efficacy and number of complications. We used an indirect assessment, based on the correct positioning of the needle, assuming that this could be linked to different outcomes in real patients. When we applied the binomial model for dependent data, we observed an increase in the chance of carrying out an appropriate injection during the training (odds ratio = 1.62; 95% CI: 1.28 to 2.05). The number of appropriate injections increased significantly from phase 1 to phase 2 (p = 0.007). In fact, there were no inappropriate injections carried out in phase 2.

After the eighth procedure, the performance of the residents was similar to that of the experienced radiologist in terms of the quality of the procedure. Although the experienced radiologist still outperformed most of the residents in terms of the parameters procedure time and fluoroscopy time, there was great improvement among the latter, particularly during phase 1, and the learning curves tended toward stability in the last procedures, resembling what was observed for the experienced radiologist. We propose that after the eighth procedure, there is little benefit in continuing the simulation-based training, and that the training could thereafter involve supervised real procedures.

Our simulator might assist in the training of facet joint injections. It is a simple, inexpensive model and can easily be constructed at any facility. We propose that it be used in interventional radiology training programs, to help beginning residents gain confidence and experience before they attempt the procedures in real patients.

## CONCLUSION

We present a simple, inexpensive simulator that can be used in interventional radiology training programs for training residents in the administration of facet joint injections. The learning curves of our volunteers showed improvement in all of the parameters evaluated (procedure time, fluoroscopy time, and quality of the procedure).

## References

[1] Peh W (2011). Image-guided facet joint injection. Biomed Imaging Interv J.

[2] Fritz J, Niemeyer T, Clasen S (2007). Management of chronic low back pain: rationales, principles, and
targets of imaging-guided spinal injections. Radiographics.

[3] Boswell MV, Trescot AM, Datta S (2007). Interventional techniques: evidence-based practice guidelines in
the management of chronic spinal pain. Pain Physician.

[4] Bogduk N (1997). International Spinal Injection Society guidelines for the
performance of spinal injection procedures. Part 1: zygapophysial joint
blocks. Clin J Pain.

[5] Silbergleit R, Mehta BA, Sanders WP (2001). Imaging-guided injection techniques with fluoroscopy and CT for
spinal pain management. Radiographics.

[6] Lilius G, Harilainen A, Laasonen EM (1990). Chronic unilateral lowback pain. Predictors of outcome of facet
joint injections. Spine (Phila Pa 1976).

[7] Marshall MB (2012). Simulation for technical skills. J Thorac Cardiovasc Surg.

[8] Ericsson KA (2004). Deliberate practice and the acquisition and maintenance of expert
performance in medicine and related domains. Acad Med.

[9] Ramsay CR, Grant AM, Wallace SA (2001). Statistical assessment of the learning curves of health
technologies. Health Technol Assess.

[10] Wanzel KR, Ward M, Reznick RK (2002). Teaching the surgical craft: from selection to
certification. Curr Probl Surg.

[11] Watson DI, Baigrie RJ, Jamieson GG (1996). A learning curve for laparoscopic fundoplication. Definable,
avoidable, or a waste of time?. Ann Surg.

[12] Hernandez J, Ross S, Morton C (2010). The learning curve of laparoendoscopic single-site (LESS)
cholecystectomy: definable, short, and safe. J Am Coll Surg.

[13] Qiu Z, Sun J, Pu Y (2011). Learning curve of transumbilical single incision laparoscopic
cholecystectomy (SILS): a preliminary study of 80 selected patients with
benign gallbladder diseases. World J Surg.

[14] Schijven MP, Jakimowicz J (2004). The learning curve on the Xitact LS 500 laparoscopy simulator:
profiles of performance. Surg Endosc.

[15] Coderre S, Anderson J, Rostom A (2010). Training the endoscopy trainer: from general principles to
specific concepts. Can J Gastroenterol.

[16] Hyltander A, Liljegren E, Rhodin PH (2002). The transfer of basic skills learned in a laparoscopic simulator
to the operating room. Surg Endosc.

[17] Rogers DA, Elstein AS, Bordage G (2001). Improving continuing medical education for surgical techniques:
applying the lessons learned in the first decade of minimal access
surgery. Ann Surg.

[18] Abdala N, Oliveira RAS, Alves Junior JDC (2007). Manikin-type training simulator model for transpedicular puncture
in percutaneous vertebroplasty. Radiol Bras.

[19] Gould D (2010). Using simulation for interventional radiology
training. Br J Radiol.

[20] Johnson SJ, Guediri SM, Kilkenny C (2011). Development and validation of a virtual reality simulator: human
factors input to interventional radiology training. Hum Factors.

[21] Lundberg J, Jonsson S, Holmin S (2010). New endovascular method for transvascular exit of arteries and
veins: developed in simulator, in rat and in rabbit with full clinical
integration. PLoS One.

[22] Gailloud P, Muster M, Piotin M (1999). In vitro models of intracranial arteriovenous fistulas for the
evaluation of new endovascular treatment materials. AJNR Am J Neuroradiol.

[23] Seymour NE, Gallagher AG, Roman SA (2002). Virtual reality training improves operating room performance:
results of a randomized.double-blinded study. Ann Surg.

[24] Newton PO, Shea KG, Granlund KF (2000). Defining the pediatric spinal thoracoscopy learning curve:
sixty-five consecutive cases. Spine (Phila Pa 1976).

[25] Jones DP, Robertson PA, Lunt B (2000). Radiation exposure during fluoroscopically assisted pedicle screw
insertion in the lumbar spine. Spine (Phila Pa 1976).

[26] Tay CW, Shen L, Hartman M (2013). SILC for SILC: single institution learning curve for
single-incision laparoscopic cholecystectomy. Minim Invasive Surg.

[27] Kasasbeh ES, Parvez B, Huang RL (2012). Learning curve in transradial cardiac catheterization:
procedure-related parameters stratified by operators' transradial
volume. J Invasive Cardiol.

[28] Blair B, Huang G, Arnold D (2013). Reduced fluoroscopy protocol for percutaneous nephrostolithotomy:
feasibility, outcomes and effects on fluoroscopy time. J Urol.

